# The Molecular Ecology of the Extinct New Zealand Huia

**DOI:** 10.1371/journal.pone.0008019

**Published:** 2009-11-25

**Authors:** David M. Lambert, Lara D. Shepherd, Leon Huynen, Gabrielle Beans-Picón, Gimme H. Walter, Craig D. Millar

**Affiliations:** 1 Griffith School of Environment and School of Biomolecular and Physical Sciences, Griffith University, Nathan, Australia; 2 Allan Wilson Centre for Molecular Ecology and Evolution, Massey University, Auckland, New Zealand; 3 Institute of Natural Sciences, Massey University, Auckland, New Zealand; 4 School of Biological Sciences, The University of Queensland, Brisbane, Australia; 5 Allan Wilson Centre for Molecular Ecology and Evolution, University of Auckland, Auckland, New Zealand; University of Poitiers, France

## Abstract

The extinct Huia (*Heteralocha acutirostris*) of New Zealand represents the most extreme example of beak dimorphism known in birds. We used a combination of nuclear genotyping methods, molecular sexing, and morphometric analyses of museum specimens collected in the late 19^th^ and early 20^th^ centuries to quantify the sexual dimorphism and population structure of this extraordinary species. We report that the classical description of Huia as having distinctive sex-linked morphologies is not universally correct. Four Huia, sexed as females had short beaks and, on this basis, were indistinguishable from males. Hence, we suggest it is likely that Huia males and females were indistinguishable as juveniles and that the well-known beak dimorphism is the result of differential beak growth rates in males and females. Furthermore, we tested the prediction that the social organisation and limited powers of flight of Huia resulted in high levels of population genetic structure. Using a suite of microsatellite DNA loci, we report high levels of genetic diversity in Huia, and we detected no significant population genetic structure. In addition, using mitochondrial hypervariable region sequences, and likely mutation rates and generation times, we estimated that the census population size of Huia was moderately high. We conclude that the social organization and limited powers of flight did not result in a highly structured population.

## Introduction

Huia (*Heteralocha acutirostris*) had the most extreme sex-linked bill dimorphism known in birds [1]–[3]. Male Huia were thought to have short, stout bills, whereas females were characterised by long curved bills, about a third longer than those of males. Males and females had such distinctive bill morphologies that they were originally described as different species [4], [5]. Observations by early naturalists suggested that the species was territorial, that juveniles lived with adults, and that family members cooperated in foraging [6].

The closest relatives of the Huia are the North Isand Saddleback (*Philesturnus carunculatus*) and the North Island Kokako (*Callaeas cinereus*). Like the Huia, these species are also wattlebirds and are characterised by a pair of colourful, fleshy wattles; strong feet; and short, rounded wings [7]. The pre-human distribution of Huia bones in caves, dunes, and middens indicates that they were once common throughout the North Island of New Zealand but were absent from the South Island [8]. Following Polynesian settlement, the species declined. Further reduction ensued as hunting pressure increased, partly because Huia tail feathers became fashionable among Europeans, especially after the Duke of York (later King George V) wore one in his hatband. Huia bills were also commonly used as brooches and these became increasingly popular in the late nineteenth century. Increased hunting, clearance of lowland forest, and the introduction of predators finally led to the Huia's extinction. The last confirmed sighting was in 1907 [9]; however, evidence suggests that the species survived until the 1930s [9 ; Lovis pers. comm.].

Little is known about the behaviour or social structure of Huia, apart from limited observations made by early naturalists. Buller [6] observed that Huia inhabited thick forest and moved mainly on foot ‘by a series of bounds or jumps’. Colenso [10] suggested that Huia were social birds, and Buller [6] noted that they were almost always found in pairs and sometimes in groups of four or more. Potts [11] observed Huia young accompanying what he assumed to be their parents for a considerable time after fledging. He gave an account of four juveniles, barely distinguishable from adults, still being fed by their parents. Moorhouse [12] suggested that Huia were highly territorial based on Buller's [6] observation that pairs were attracted by imitations of their call, a trait also common to the Saddleback and Kokako [7]. Their social organisation and limited powers of flight suggests that Huia are likely to have exhibited a high level of population genetic structure.

**Figure 1:**
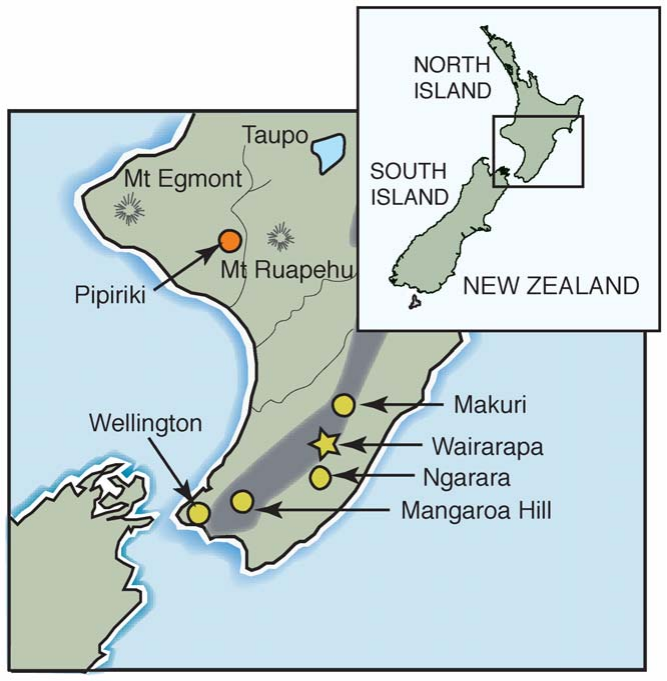
Provenance of Huia samples used in this study. Precise sample locations are indicated by circles and approximate locations by stars. Other place names are for reference only.

We examined museum samples collected from both sexes and from a number of locations (Figure 1) to understand the population genetic structure of Huia and the nature of their sexual dimorphism. We used several nuclear genotyping markers isolated from extant Saddleback to amplify ancient DNA from Huia. Using rigorous ancient DNA methodologies, we were able to determine the genotypes of a number of individuals unambiguously. To determine the relationship between bill morphology and sex in Huia, we used molecular sexing methods and correlated these results with morphometric measurements.

## Results

**Figure 2:**
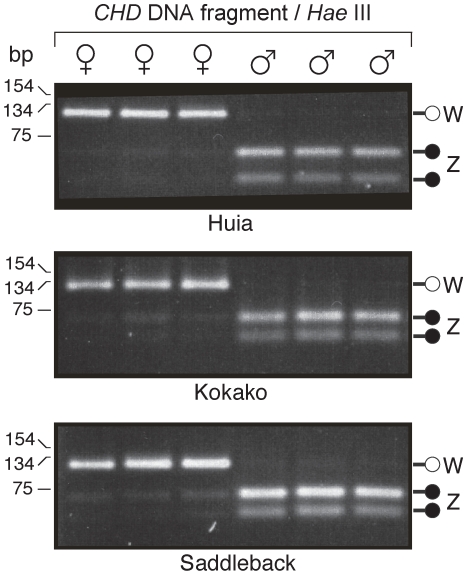
Huia sexing using semi-nested PCR with *CHD* primers p2/p5 followed by p2/p3. Amplification was followed by digestion of the Z *CHD* fragment with the restriction enzyme *Hae*III. The W chromosome *CHD* fragment in Huia is 121 bp long and the Z chromosome fragments are 65 and 56 bp in length, after enzyme digestion. Known-sex relatives of Huia, the North Island Kokako and Saddleback, were used to verify the test. Molecular weight maker sizes at shown at left.

**Figure 3:**
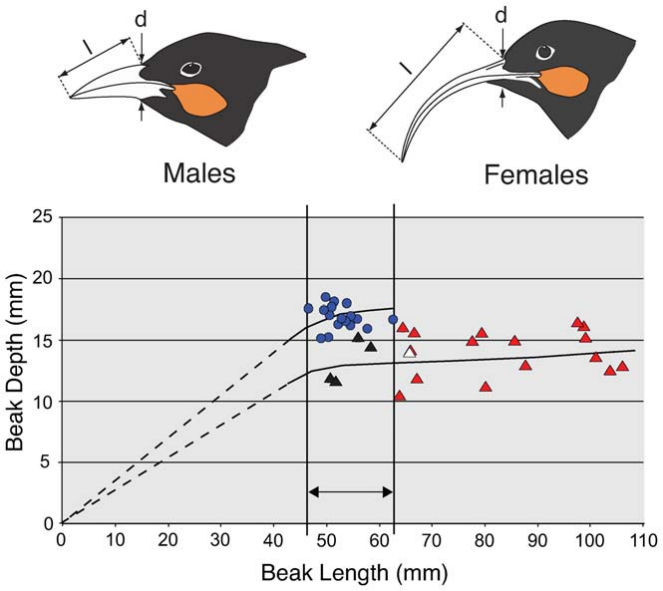
The relationships between beak length and depth for 17 male and 21 female Huia. Arrows indicate where measurements were made. Sex was determined using molecular methods. Blue circles represent males, red triangles indicate females, and black triangles represent the four DNA-sexed females that fall within the beak length range of males. Another DNA-sexed female, which was labelled ‘juvenile’, is indicated by a white triangle. Possible growth curves for males and females are shown by the black lines.

We determined the molecular sex of 38 Huia specimens, as described in the Methods section. We show that Z and W chromosome sequences can be amplified in Huia and, along with digestion of these products with the restriction enzyme *Hae* III, allowed ZZ males to be distinguished from ZW females (Figure 2). Discrimination of the sexes was aided by the preferential amplification of the W *CHD* locus which was also recorded for Huia's close relatives, the Kokako and the Saddleback. We routinely find this with other avian sexing work (unpublished data), especially when using nested PCRs. This preferential W loci amplification fortuitously provided us with very clear sexing results – either W loci amplification only, or just Z loci amplification. Of the 38 specimens successfully sexed, 17 were shown to be males and 21 were females. Morphometric data show that Huia males had an average beak length of 52.8 mm, whereas female beaks were typically much longer, with an average of 77.9 mm. This difference was highly significant using a two-tailed t-test 22 df, assuming unequal variance (P<0.0001). In contrast, the mean female beak depth (13.9 mm) was significantly smaller than that of males (16.9 mm) using a two-tailed t-test 33 df and assuming unequal variance (P<0.0001). Variation in the beak length of males was relatively small (SD ±3.8 mm), while in females it was much larger (SD ±18.2 mm). To test the null hypothesis that there was no difference in the variance between male and female beak lengths, we conducted a one-tailed *F*-test. The test statistic is the ratio of two sample variances, and the difference was highly significant 20, 16 df (P<0.0001). In addition to beak length, females showed a significantly greater variance in beak depth (one-tailed *F*-test 20, 16 df; P = 0.012). Moreover, four DNA-sexed females were indistinguishable from males in terms of beak length (Figure 3; these individuals are indicated by black triangles). Another DNA-sexed female was intermediate between the males and shorter beaked female Huia (Figure 3), and was labeled as a “juvenile” in the museum collection (white triangle).

### Genotyping

Eighteen of twenty-five Huia samples (72%) amplified for four or more of the six polymorphic microsatellite DNA loci used. All loci were in Hardy-Weinberg equilibrium (Table 1). No linkage was observed between loci following adjustment of the significance level for multiple comparisons with a Bonferroni correction [13]. Loci exhibited moderate levels of variation with 2–10 alleles per locus, and expected heterozygosities ranged from 0.437–0.766, with a mean of 0.637 across all loci (Table 1). The mean number of loci amplified per sample was 5.7 of the 6 loci. The mean shared allele distance between presumably non-related Huia was calculated to be 0.553 across all Huia individuals, except those in a putative family (AV2745, AV2746, and AV2747; Table 2).

**Table 1:** Genetic diversity measures and genotyping errors at six microsatellite DNA loci amplified from Huia.

Locus	Pca01	Pca05	Pca12	Pca13	Pca16	K9/K10	Overall
Number of alleles (N_A_)	4	3	10	2	5	6	30
Allele size range (bp)	178–186	131–135	113–130	157–159	114–127	69–85	-
H_O_	0.714	0.583	0.750	0.348	0.762	0.818	0.662
H_E_	0.692	0.494	0.766	0.437	0.713	0.723	0.637
Hardy-Weinberg p values	0.201	0.967	0.777	0.896	0.610	0.649	-
Allelic dropout: Longer allele missing	6	0	6	2	2	4	20
Allelic dropout: Shorter allele missing	1	1	0	2	1	2	7
ADO_μ_	0.179	0.032	0.133	0.200	0.088	0.146	0.129
FA_μ_	0.024	0.019	0	0	0	0	0.007

The number of alleles (N_A_), their size ranges, observed and expected heterozygosities and genotyping errors (allelic dropout rate: ADO_μ_, and rate of occurrence of false alleles: FA_μ_) are given.

**Table 2:** Huia microsatellite DNA genotypes obtained following the ‘multiple tubes’ approach.

	Likely Genotype at Locus					
Sample Name	Pca01	Pca05	Pca12	Pca13	Pca16	K9/K10
AV2727	182/182 (7/7)	135/133 (2/2)	121/119 (2/2)	157/157 (7)	-	81/72 (2/3)
						*81/81 (1/3)*
AV37493A	184/182 (2/3)	135/133 (2/2)	121/119 (2/2)	157/157 (7/7)	121/119 (1/4)	72/72 (7/7)
	**186/184/182 (1/3)**				*119/119 (1/4)*	
					*121/121 (2/4)*	
AV1083	186/182 (2/3)	135/133 (2/2)	119/115 (2/3)	157/157 (7/7)	119/113 (2/2)	72/83 (1/3)
	*186/186 (1/3)*		*115/115 (1/3)*			*72/72 (1/3)*
						*83/83 (1/3)*
AV21283	Not consistently scorable	133/133 (7/7)	130/116 (2/2)	157/157 (7/7)	-	72/72 (7/7)
HBH	186/186 (7/7)	135/133 (3/3)	119/115 (3/3)	157/157 (7/7)	121/117 (2/2)	72/72 (7/7)
AV374934B	184/182 (2/2)	135/133 (2/2)	121/115 (2/2)	159/157 (2/2)	-	81/72 (2/2)
AV2745*	184/184 (7/8)	133/133 (8/9)	121/119 (3/3)	157/157 (7/7)	119/117 (2/2)	85/72 (2/2)
	**186/184 (1/8)**	**133/131 (1/9)**				
AV2244	-	133/131 (2/3)	119/115 (2/3)	-	117/117 (7/7)	85/72 (2/2)
		*133/133 (1/3)*	*115/115 (1/3)*			
AV2747*	184/182 (2/4)	135/133 (2/2)	121/119 (2/2)	159/157 (2/2)	119/119 (7/7)	85/72 (2/2)
	*182/182 (2/4)*					
AV2283	186/178 (2/2)	135/135 (7/7)	121/115 (2/2)	159/157 (2/4)	117/117 (7/7)	81/81 (7/7)
				*157/157 (2/4)*		
AV2744	184/182 (2/3)	135/133 (2/2)	122/115 (2/4)	159/157 (2/2)	121/119 (2/2)	85/72 (2/3)
	*182/182 (1/3)*		*115/115 (2/4)*			*72/72 (1/3)*
AV2746*	186/182 (2/3)	135/135 (7/7)	121/115 (2/2)	159/159 (7/7)	121/119 (2/2)	72/69 (2/2)
	*182/182 (1/3)*					
AV1082	184/178 (2/2)	135/133 (2/2)	129/121 (2/2)	157/157 (7/7)	117/113 (2/2)	83/72 (1/3)
						*83/83 (1/3)*
						*72/72 (1/3)*
AV2245	-	133/133 (8/8)	119/117 (3/3)	159/157 (2/4)	121/119 (2/2)	74/69 (2/3)
				*159/159 (2/4)*		*69/69 (1/3)*
AV1078	184/182 (2/2)	135/133 (2/2)	115/113 (2/2)	157/157 (7/7)	121/117 (2/2)	81/74 (2/2)
AV1085	186/184 (2/2)	133/133 (7/7)	130/115 (2/2)	159/157 (2/2)	121/119 (2/2)	-
AV21289	186/184 (2/2)	133/133 (7/7)	115/115 (7/7)	157/157 (7/7)	121/117 (2/2)	85/72 (2/2)
AV36838	184/182 (2/3)	133/133 (7/7)	126/121 (2/4)	157/157 (7/7)	127/119 (2/2)	81/72 (2/2)
	*182/182 (1/3)*		*121/121 (2/4)*			

False alleles are shown in bold text. False genotypes resulting from allelic dropout are shown in italic text. – indicates no amplification; * indicates three members of the same putative family.

### Genotyping Error

Data for consensus genotypes are presented in Table 2. The ADO_μ_ (frequency of allelic dropout) of each locus varied from 3.2% to 20% (Table 1) with a mean of 12.9%. Seven replications were carried out for each locus so that the probability of obtaining false homozygotes was negligible (P<0.0001). Across all loci, the longer of the two alleles in a heterozygote was significantly more likely to not amplify (20 longer alleles verses 7 shorter alleles ‘dropped out’; Chi squared test χ^2^ = 6.24, d.f. = 1, p = 0.01). Allelic dropout rates also differed significantly among samples (Chi squared test χ^2^ = 36.11, d.f. = 23, p = 0.040). Three false alleles were observed in the dataset (rate of occurrence  = 0.7%).

Allelic dropout is thought to be a significant problem for genetic census data from degraded DNA samples such as those analysed here. This can result in false genotypes and hence can cause overestimation of population size [14]. In contrast, the effect of allelic dropout on estimations of population structure is probably less of a problem [15], although it has not been rigorously investigated [16]. The microsatellite genotyping error rates determined for the Huia dataset are within the range of those encountered in other studies of samples with low template concentrations [reviewed in 17]. Allelic dropout was a more common form of genotyping error in this dataset than the occurrence of false alleles. The probability of false homozygotes at each locus owing to allelic dropout was calculated to be negligible after seven replicates were averaged for each sample. However, this conclusion is based on the assumption that all individuals have equal dropout rates. This is probably violated in most datasets. Consequently, a few undetected dropouts may remain in the Huia dataset. However, the number is likely to be minimal because low quality samples were identified and removed prior to analysis. This approach has been found to be reliable in decreasing genotyping errors in other studies [14], [18].

### Estimating Genetic Diversity and Population Size of Huia

**Figure 4:**
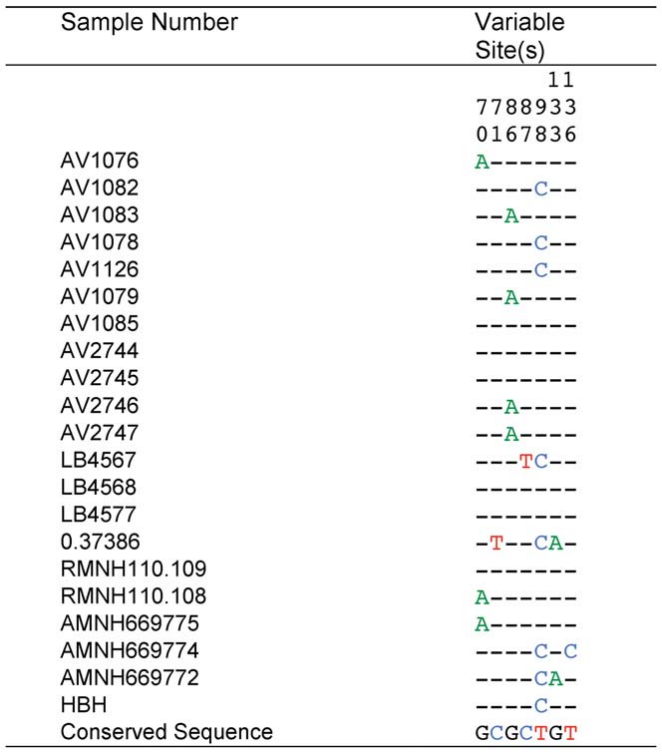
DNA nucleotide variation in 199 bp of the mitochondrial hypervariable region (HVRI) among 21 Huia.

We recorded eight mitochondrial haplotypes among the 21 Huia individuals examined (Figure 4). The resulting dataset did not show extensive levels of artifactual mutations; samples 0.37386 and LB4568 only have two C>T singletons that could have arisen as a result of post mortem deamination of cytosine residues. If artifactual, these singletons would lead to a false overestimation of the extent of population expansion, and would consequently provide false census size estimates. However, these two samples were sequenced in the forward and reverse direction from independent amplifications and both sequence reads cover the singletons in question. Therefore, we are confident that they represent real variation. We used these data to obtain an estimate of genetic diversity (θ) of 0.011846 (95% credibility interval 0.005804–0.031121) from 199 bp of mitochondrial hypervariable region sequence. Mitochondrial data were used in this analysis because mutation rates for the hypervariable region are better known. The relationship between θ and the effective population size of breeding females is given by the expression N_e_(f) = θ/*2μ*, where μ is the mutation rate per base pair per generation and generation time is defined as the average age at which a female reproduces. A generation time of 6.3 years was used in our analyses, and was based on the generation times of the related North Island Kokako [19] and Saddleback [20]. We used a mutation rate of 5.5×10^−7^ mutations per site per year for the hypervariable region, based on a pedigree study in Adélie penguins [21], as well as the phylogenetic estimate of 2.1×10^−7^ mutations per site per year. The former resulted in a mutation rate per generation of 3.47×10^−6^ for Huia and the latter 2.65×10^−7^. Using the above values, N_e_(f) for Huia was estimated at 1709–4477. The range of estimates of the overall effective population size (N_e_) of Huia using this generation time and these mutation rate variables was 3419–8954 breeding adults, assuming an equal sex ratio. The ratio of effective to census population size (N_c_) in wild populations is often quite low and it has been suggested that a N_e_: N_c_ ratio of 0.1 is appropriate [22]. This resulted in an expected census population size of 34,187 birds for the higher mutation rate. If we use the slower molecular rate of 2.1×10^−7^ [23], the mean estimate of the census population size is 89,538. Table 3 gives details of the credibility intervals for all these estimates. This range of population size estimates is best described as ‘moderately high’. Estimates of growth rate (g) were consistently positive and large (∼1111), with confidence intervals excluding zero (419–2776), indicative of an expanding population (http://evolution.gs.washington.edu/lamarc/).

**Table 3:** Long-term population size estimates of Huia based on mitochondrial hypervariable region diversity.

Mutation rate per base pair per generation (6.3 years)	Genetic diversity θ mean (95% credibility interval)	N_e_(f) (θ/2μ)	N_e_ (N_e_(f) ×2)	N census size (N_e_∶N_c_ ratio of 0.1)
**3.47×10−6**	**0.011846** (0.005804–0.031121)	**1709** (838–4491)	**3419** (1675–8982)	**34188** (16750–89815)

### Huia–Saddleback Comparisons

Huia exhibited a greater mean number of alleles per locus than did its relative, the North Island Saddleback, when polymorphic loci alone were considered (Table 4). Huia also had a higher mean number of alleles per locus when all loci that were polymorphic in either Huia or Saddleback (i.e., all loci in Table 4) were measured. The mean expected heterozygosity was higher in Huia (0.637) than in Saddleback (0.559, [24] and the length distribution of alleles at each locus was more continuous. For example, at locus Pca12, Huia possessed ten alleles differing in size by 1–4 bp, whereas only two alleles were detected in Saddleback, which differed by 16 bp.

**Table 4:** The number of alleles (N_A_) at microsatellite DNA loci isolated from North Island Saddleback that are polymorphic in North Island Saddleback and/or Huia.

Locus	N_A_	
	Saddleback	Huia
Pca01	1	4
Pca02	4	1
Pca05	3	3
Pca08	3	1
Pca10	2	1
Pca12	2	10
Pca13	1	2
Pca14	3	1
Pca15	3	1
Pca16	1	5
Mean N_A_/locus ± SE	2.30±0.33	3.00±0.91
Mean N_A_/polymorphic locus ± SE	2.86±0.26	5.00*±1.15

*The mean N_A_/polymorphic locus in Huia includes locus K9/K10 isolated from Kokako.

Huia averaged a greater number of alleles per locus over all polymorphic loci than Saddleback, despite fewer Huia samples being genotyped. Moreover, Huia had higher levels of heterozygosity than the Hen Island Saddleback population, from which all contemporary populations derive [24]. These results are unexpected because microsatellite loci are commonly longer and more variable in the species from which they are derived [25]. However, this is consistent with the fact that North Island Saddleback lost a considerable portion of their genetic variation through a population bottleneck [24].

## Discussion

We suggest that the large variation in bill length and depth of female Huia, along with our molecular sexing data, indicate that female Huia may have began life with short stout bills indistinguishable from those of males and that their beaks grew longer over their lifetime. The primary evidence for this is that we recorded four female Huia that were DNA sexed, and these individuals had bills indistinguishable from males in terms of length. In addition a single Huia specimen labeled as a ‘juvenile’ was sexed as a female but had a very short bill. Several processes could explain the greater coefficient of variation in female bill length. For example, bill length might be controlled by W chromosome-specific loci and females consequently show much greater variation, or alternatively female bill growth may have been influenced by other factors such as diet. There is evidence for both these processes in avian species [26], [27].

Our data show that Huia were characterised by a high level of genetic variation. Two genetic studies of Kokako reported low levels of genetic structuring among modern populations for both mtDNA [19] and microsatellite DNA loci [28]. However the level of genetic variation in Kokako, prior to their reduction in numbers, remains unknown. Although Kokako adults remain in the same territory for many years, it has been suggested that juveniles disperse early to find mates and/or establish territories [29], and that this is responsible for the recorded low levels of genetic structure. In contrast, early observations of Huia behaviour, such as apparent site fidelity and the presence of family groups, suggest that Huia would exhibit a high level of population genetic structure. To test this idea, we examined six polymorphic nuclear loci in Huia. Since many of the museum specimens examined in this study did not have detailed location data, a series of analytical methods were used to detect population differentiation independent of geographic information. The most likely number of populations in the Huia dataset of genotypes was estimated using a Bayesian clustering approach implemented in STRUCTURE [30]. This analysis suggested no evidence of population differentiation among the Huia samples examined (Table 5). Population structure was also investigated using PARTITION [31]. Using this algorithm, the tree plot of Huia genotype data also provided no evidence of population subdivision, as no well-supported clusters were separated by long branches. The plot of Bayesian probability levels versus generation time also indicated no evidence of population structure, i.e. the probability level declined gradually to a single cluster of individuals. Both analyses suggest a lack of population genetic structure in Huia, despite known provenance samples coming from locations up to 300 km apart. However, the area from which these samples came was covered in continuous lowland forest prior to human settlement, which may have allowed extensive mixing of resident populations.

**Table 5:** Estimated posterior probabilities, P(K/X), of K, the number of Huia populations.

K	lnP(X/K)	P(K/X)
1	−335.2	0.925
2	−338.1	0.051
3	−339.9	0.009
4	−339.4	0.014
5	−343.8	0.002

The estimated probability of the data, lnP(X/K), averaged over four independent runs for each K.

Our estimate of the population size of Huia is relevant to the period prior to the human settlement of New Zealand and, depending on likely mutation rates and generation times, indicates a ‘moderate’ historical population size of 34,187 to 89,539 individuals. It is likely, however, that the pre-human population size was higher than our estimate, as our analyses show a period of population expansion. In combination, these factors suggest moderate to high Huia population densities. As a consequence, gene flow within the population is likely to be significant and will thereby promote genetic homogeneity within the species. In addition, populations of moderate size, such as that of Huia, typically exhibit a level of genetic inertia that may also have contributed to the overall genetic homogeneity of the species. This finding is in contrast to our original expectation that Huia populations would have exhibited high levels of population structure.

In conclusion, our molecular sexing results provide evidence that female Huia bills develop from a male-like condition. We suggest that young females may have been indistinguishable from males in terms of bill length, and that the typical differences developed over the lifetime of the individuals. Mitochondrial DNA variation in Huia allowed us to estimate the likely historical census population size for this species. Despite the suggestions from early naturalists that Huia were highly territorial and that the species had limited dispersal capability, no evidence for population genetic structure was detected using a range of nuclear loci. Generally, this work illustrates the potential of both sex-linked and autosomal DNA sequences to improve understanding of the population dynamics and molecular ecology of an extinct species. Furthermore, as many species of conservation concern become rarer in the wild, scientific programmes might benefit from using older specimens in museums. This would have less impact on modern populations of these species and would make use of a valuable museum resource. Hence, similar ancient DNA methods to those used here could be applied to scientific attempts to better understand the causes of species' decline.

## Materials and Methods

### Samples

Twenty-four Huia footpad samples were provided by the Canterbury Museum, 13 from the Auckland Museum, seven from the Naturalis Museum (Leiden), 24 from the American Museum of Natural History, two from Macleay Museum, two from the Australian Museum, and one sample from a private collector (Table 6). Exact provenance data were not known for 46 of these samples; the collection locations of the eight samples used for microsatellite analysis, plus two labelled as ‘possibly Pipiriki’, are illustrated in Figure 1.

**Table 6:** Huia samples used in this study.

Museum Number	Location	Collector and Presentation Date	Morphological Sex	Molecular Sex (# times sexed)	Beak	
					L	D
AV1076	Wairarapa	Buller, 1891	Female	Female (2)	99.03	16.17
AV1078	Makuri	1892	Male	Male (1)	55	17
AV1079	Ngarara	Buller, 1891	Male	Male (1)	54.01	18.16
AV1081	-	-	Female	Female (1)	77.83	14.98
AV1082	Wairarapa	Buller, 1892	Male	Male (1)	50.09	18.61
AV1083	-	-	Female	Female (1)	51	11.9
AV1085	-	-	Male	Male (3)	50.48	17.3
AV1087	-	Moorhouse	Male	Male (3)	50.85	17.69
AV1126	-	-	Male	Male (2)	52.5	16.44
AV2244	-	Parker	Female	Female (1)	85.93	14.95
AV2245	-	Parker	Male	Male (1)	46.8	17.71
AV2283	-	Parker	Male	Male (1)	55.44	16.94
AV2727	-	Codmor	Female	Female (1)	67.35	11.9
AV2729	-	O'Connor	Male	Female (5)	56.16	15.27
AV2744	Wellington	-	Male	Male (3)	54.71	16.33
AV2745	Mangaroa Hill	Len Harris, 1885	Male	Male (1)	51.29	18.11
AV2746	Mangaroa Hill	Len Harris, 1885	Female	Female (3)	87.98	13
AV2747	Mangaroa Hill	Len Harris, 1885	Female (juvenile)	Female (2)	66.2	14.13
AV21283	-	P. Hall	Male	Male (1)	49.78	17.52
AV21289	-	-	Female	Female (2)	106.4	12.93
AV36838	-	F.Grimwood, 1870s Gifted by a North Island Maori Chief	Female	Female (3)	79.65	15.68
AV37493A	Possibly Pipiriki	Mrs F. Stewart	Male?	Female (2)	64	10.5
AV37493B	Possibly Pipiriki	Mrs F. Stewart	Female	Female (4)	104	12.5
HBH	-	-	Female	Female (2)	66	14
AV1070	-	-	Female	Female (2)	52	11.7
LB4564	North Island	-	Male	Male (3)	62.7	16.3
LB4565	North Island	-	Male	Female (1)	68.0	15.5
LB4567	Ruahine Range	C.E. Clarke, 20 Aug 1931	Male	Male (1)	58.2	15.9
LB4568	Ruahine Range	C.E. Clarke, 20 Aug 1931	Female	Female (1)	99.2	16.5
LB4571	North Island	S.H. Mountford, 1941	Male	Male (1)	54.3	16.5
LB4572	North Island	S.H. Mountford	Male	Male (1)	51.0	15.3
LB4573	North Island	S.H. Mountford	Female	Female (1)	64.2	15.8
LB4575	North Island	S.H. Mountford	Female	Female (1)	59.5	14.3
LB4576	North Island	C.A. Fleming	Female	Female (1)	80.3	11.7
LB4577	North Island	C.A. Fleming	Male	Male (1)	52.0	16.8
LB9213	-	J.A. Rentoul, 03 Dec 1969	Female	Female (1)	100.7	13.7
LB9215	-	-	Male	Male (1)	49.0	15.2
LB9217	-	-	Female	Female (3)	99.5	15.21
RMNH110.108	Rimutaka, Hills, Wellington	Travers, 1898	Male			
RMNH110.109	Rimutaka Hills, Wellington	Travers, 1898	Female			
AMNH669772	Wellington	1892	Male			
AMNH669774	Makuri Ranges		Male			
AMNH669775	Makuri Ranges		Male			

Museum numbers and presentation details if known and sex (morphological and molecular) are given. HBH  =  specimen obtained from Hastings Boys' High School; RMNH  =  Rijksmuseum van Natuurlijke Historie; AMNH  =  American Museum of Natural History, Beak length (L) and depth (D) are measured in millimetres.

### Ancient DNA Methods

Extraction of ancient Huia DNA was performed in a dedicated ancient DNA laboratory physically separated from where contemporary DNA and PCR products were handled. Decontamination was routinely carried out by UV-irradiation and sodium hypochlorite washes. Approximately two mm^2^ of Huia footpad tissue was removed and cut into several pieces using a sterile razor blade. Huia DNA was extracted by incubating footpad fragments overnight at 50°C in 2.5 ml of extraction buffer (10 mM Tris-HCl pH 8.0, 10 mM NaCl, 10 mM EDTA), 250 µl of 10% SDS, 15 µl of 200 mg/ml dithiothreitol (DTT), and 25 µl of 50 mg/ml Proteinase-K. Samples were then extracted with phenol followed by chloroform: isoamyl alcohol (24∶1) and then concentrated by centrifugation through a Vivaspin-30 (Viva Science, U.K.) membrane. Negative extraction controls were included with every 6–12 sample extractions. All mitochondrial sequences were obtained by PCR as outlined below and sequenced in the forward and reverse direction from multiple independent amplifications. Huia HVRI sequences are deposited in the GenBank database with accession numbers GU176413–GU176433.

### Huia Mitochondrial Hypervariable Region Sequences

One hundred and ninety-nine bp of the Huia hypervariable region I (HVRI) region were amplified from 21 individuals (Figure 4) as outlined below using primers huiaIF (5′-ATAAACCCAAGTGATCCTACCT) and huiaIIR (5′-TTGAGTAGCTCGGTTCTCGTGA). Amplification products were purified by centrifugation through Sephacryl^TM^ S200HR (GE Healthcare), sequenced using ABI BigDye® Terminator v3.1 chemistry, and analysed using an ABI 3730 Genetic Analyzer. Sequences were edited and aligned in Sequencher^TM^ 4.6 (Gene Codes Corporation).

### Molecular Sexing of Huia and Morphometrics

The following primers were used to amplify Z and W chromosome sequences in Huia and thereby distinguish ZZ males from ZW females: p2, p3 [32], and a novel primer designed for Huia p5 (5′-GTAGGAGCAGAAGATATTCTG). These amplify a region of the sex-linked chromodomain-helicase-DNA gene (*CHD*) [32; Figure 2]. Amplifications were carried out in 10 µl reaction mixes containing 50 mM Tris-HCl pH 8.8, 20 mM (NH_4_)_2_SO_4_, 1 mg/ml bovine serum albumin (BSA), 2.5 mM MgCl_2_, 100 µM of each dNTP, 0.3 U of AmpliTaq® DNA Polymerase (Perkin Elmer), 40 ng of each primer, and approximately 1 ng of DNA. DNA was amplified using a Hybaid OmniGene thermal cycler. The initial amplification profile was 1×94°C 2 min, then 10×94°C for 20 sec, 55°C for 20 sec, and 72°C for 20 sec followed by 35×94°C for 20 sec, 52°C for 20 sec, and 72°C for 20 sec. A second PCR was carried out by adding approximately 0.3 µl from the initial amplification mix to a fresh reaction mix. This mix was then cycled as follows: 1×94°C 2 min then 10×94°C for 20 sec, 55°C for 20 sec, and 72°C for 20 sec, followed by 25×94°C for 20 sec, 52°C for 20 sec, and 72°C for 20 sec. For restriction enzyme digestion, 1.2 µl of React 2 (Gibco-BRL), approximately 1 U of *Hae* III, and water was added directly to the PCR mix to a final volume of 12 µl. This mix was incubated at 37°C for 30 min before 3 µl of the digest was electrophoresed in 1.0%LE/1.5%MS agarose (Boehringer Mannheim) in TBE buffer. The gel was stained with ethidium bromide and the DNA was visualised over UV light. We also collected data on bill length and width using Vernier callipers accurate to 0.01 mm. Details of samples are provided in Table 6.

**Figure 5:**
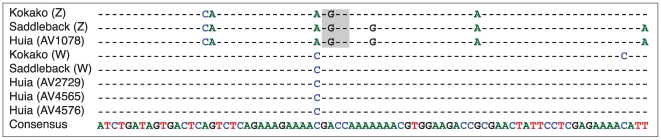
Sequence line-up of W and Z chromosome *CHD* sequences spanning the sex-specific *Hae*III restriction enzyme recognition sequence (GGCC) (grey box).

DNA products amplified with p2/p5 from samples AV2729, LB4565, AV1078, and LB4576 were cloned using a TOPO TA Cloning kit® (invitrogen) and several clones from each bird were sequenced. As expected when using these primers, only the *CHD* W allele could be isolated from the females. The *CHD* Z allele was isolated from male Huia AV1078 (Figure 5) in a similar way.

### Nuclear Genotypes of Huia

Seventeen dinucleotide microsatellite DNA loci that had been isolated from a North Island Saddleback genomic library [24; T. King & D. Lambert, unpublished data] and eight loci isolated from a Kokako microsatellite library [28], [33] were screened for polymorphism in Huia. PCR amplification was performed in 10 µl volumes containing 0.5 units of Taq polymerase (Roche), 200 µM of each dNTP, 0.8 pmol of each primer, 1.5 mM MgCl_2_, 1x PCR buffer (50 mM Tris pH 8.8, 20 mM (NH_4_)_2_SO_4_), and approximately 1 ng of extracted DNA. Samples were amplified by initial denaturation at 94°C for 4 minutes, followed by 35 cycles of 94°C for 45 seconds, then 50–55°C for 50 seconds followed by 72°C for one minute, with a final extension time of 72°C for 5 minutes. For each locus, PCR products amplified from seven Huia DNA samples were initially size fractionated in 1.0%LE/2.0%MS agarose to determine heterozygosity. Fluorescent dyes were used to label the reverse primer of each primer pair. Specifically Pca05, Pca12, Pca13, and Pca16 were labelled with 6-FAM; Pca01 with VIC; and K10 with HEX. Microsatellite DNA loci were amplified using the method described above and a negative control was included with every set of 6–12 reactions. Single-locus PCR reactions were pooled within samples where possible. Pooled reactions were genotyped using an ABI 3730 DNA Genetic Analyzer and visualised using Genescan. A sample standard was included with each genotyping run to account for between-run variation.

### Genotyping Errors

Two techniques were used to avoid genotyping errors. Firstly, an initial screen of the quality of the samples was performed from the first round of PCR reactions [14]. Samples that amplified at fewer than three of the six loci were omitted from the study. For the remaining samples, the ‘multiple tubes’ method was used [34], [35]. This approach involves multiple independent PCR amplifications of each locus to produce a consensus genotype. Putative homozygous genotypes were obtained seven times in order to discount allelic dropout (ADO_μ_) and both alleles of putative heterozygotes were detected twice in order to rule out false alleles. In addition, a subset of four Huia samples were independently extracted and amplified for three loci (Pca01, Pca05, and Pca12) at an ancient DNA facility at the University of Auckland.

### Analytical Methods

Measures of genetic variation, including observed and expected heterozygosities (H_O_ and H_E_, respectively) and number of alleles, were calculated in Arlequin 2000 (http://cmpg.unibe.ch/software/arlequin/software/2.000/doc/faq/faqlist.htm). GENEPOP version 3.4 was used to test for linkage disequilibrium and deviation from Hardy-Weinberg parameters [36]. The sequential Bonferroni correction was applied to adjust the level of significance for multiple tests [13]. False alleles can occur in heterozygous and homozygous genotypes. Therefore, the rate of false allele formation is estimated across all positive amplifications. In contrast, allelic dropout can only be detected in heterozygotes; therefore, the allelic dropout rate is calculated using only the positive amplifications of heterozygotes. The rate of allelic dropout (ADO_μ_) and the occurrence of false alleles (FA_μ_) was calculated for each locus and across all loci using the equations below, as recommended by Broquet & Petit [17] (these calculations included the genotype data independently replicated at the University of Auckland).

Allelic dropout was calculated using the equation ADO_μ_  =  D_j_/A_hetj_, where D_j_  =  the number of amplifications of locus j where an ADO event is observed, and A_hetj_  =  the number of positive amplifications of heterozygotes. The number of false alleles (FA_μ_) was determined using FA_μ_  =  F_j_/A_j_, where F_j_  =  the number of amplifications at locus j where a false allele is observed, and A_j_  =  the total number of amplifications (both hetero- and homozygotes).

The probability of false homozygotes at each locus after repeated PCR reactions (*P*) was calculated using the equation *P* = (*K*)×(*K/2*) *^n^* ^−1^ [37] where *K*  =  the ADO_μ_ at each locus and *n* is the number of repeated amplifications; in this work, n = 7.

Consensus genotypes obtained from the multiple tubes method were used to examine population structure in Huia. The lack of provenance for the majority of samples prevented the application of traditional population genetic analyses such as F-statistics. Instead, two Bayesian clustering methods that do not require prior population information to partition samples into genetic groups were used to detect any possible genetic structuring in the Huia microsatellite dataset: STRUCTURE 2.1 [30], [38] and PARTITION [31]. Neither method requires the population of origin for individual samples, or even the number of sampled populations (K) to be known. Both methods identify clusters of individuals that are in Hardy-Weinberg and linkage equilibrium, but differ in their treatment of admixed individuals [39].

STRUCTURE was used with no input of prior population information in relation to individual samples, and admixture was assumed. Allele frequencies among clusters were considered to be independent to prevent overestimation of cluster number [38]. Four independent analyses of K = 1–5 were performed using 10^6^ MCMC repetitions with the first 50,000 repetitions being discarded as ‘burn-in’ following visual confirmation that equilibrium had been reached. To select the optimal K, the posterior probabilities of the data, P(X/K) were calculated from the mean estimate log-likelihood of each K (lnP(X/K)).

PARTITION was applied only to the Huia samples that possessed a full complement of genotype data because missing data are not permitted in this software package. The parameter *μ* (the prior probability distribution on K) was set at 1, i.e. equal probabilities of each K were assumed, and the parameter θ (the prior distribution of alleles in the ancestral population) was varied from 1 to 20. The maximum number of source populations was changed with each analysis from 4 to 8. Estimates of the posterior probabilities were made after 50,000 observations of the Markov chain, with the first 5,000 observations omitted as ‘burn-in’.

Shared allele distances (1minus half the average number of shared alleles per locus) between Huia were calculated online (http://www2.biology.ualberta.ca/jbrzusto/sharedst.php). The mean number of alleles (N_A_) per locus and mean expected heterozyosity (H_E_) were compared between Huia and Saddleback. Data from 41 individuals from the Hen Island population of Saddleback [24] were used in these comparisons as all contemporary Saddleback populations derive from this island.

### Estimating Genetic Diversity and Effective Population Size in Huia

The genetic diversity parameter (θ) was calculated using a Bayesian framework [LAMARC v2.1.2; 40] that uses a coalescent approach to obtain a joint estimate of various population genetic parameters such as genetic diversity, growth, migration, and recombination rates. We estimated θ from 21 individuals for a 199 bp fragment of the mitochondrial hypervariable region. To ensure that the Bayesian estimate of θ was robust, we performed a number of repeat analyses. Fourteen preliminary analyses were conducted with a range of starting parameters (e.g. sample size and sampling increment). Posterior probability distributions were compared between runs to assess whether we converged on an estimate of θ. We also assessed convergence by calculating the effective sample size (ESS) (using the program Tracer v1.4, (http://beast.bio.ed.ac.uk/Tracer). An ESS of 100–200 has been suggested to indicate convergence. Our estimates were well above this value, in the thousands or greater. After preliminary analyses were completed, a final estimate of θ was performed from 10 replicates, each with the following starting parameters: θ = 0.015, and a linear prior of 0.0001–3. Two initial chains sampled 5000 trees with a sampling increment of 40, of which the first 7000 trees sampled were discarded, followed by 4 final chains to produce the estimate, in which, after a burn-in of 5000 trees, every 50^th^ tree of 5×10^6^ trees was sampled. Adaptive chain heating was used (chain temperatures 1, 1.1, 1.2, and 1.3). We also performed separate LAMARC analyses to test for signatures of exponential growth or shrinkage using the same searching strategy.

## References

[1] Selander RK (1966). Sexual dimorphism and differential niche utilization in birds.. Condor.

[2] Selander RK, Campbell B (1972). Sexual selection and dimorphism in birds.. Sexual Selection and the Descent of Man 1897–1971.

[3] Burton PJK (1974). Anatomy of head and neck in the huia (*Heteralocha acutirostris*) with comparative notes on other Callaeidae.. Bulletin British Museum Natural History (Zoology).

[4] Gould JA (1837). Synopsis of the birds of Australia and adjacent Islands..

[5] Lack D (1971). Ecological Isolation in Birds..

[6] Buller WL (1888). A History of the Birds of New Zealand..

[7] Heather BD, Robertson HA (1998). The Field Guide to the Birds New Zealand..

[8] Worthy TH, Holdaway RN (2002). The Lost World of the Moa..

[9] Phillips WJ (1963). The Book of the Huia..

[10] Colenso W (1887). A description of the curiously deformed bill of a huia (*Heteralocha acutirostris*, Gould) an endemic New Zealand bird.. Transactions and Proceedings of the New Zealand Institute.

[11] Potts T (1885). Oology of New Zealand.. New Zealand Journal of Science.

[12] Moorhouse R J (1996). The extraordinary bill dimorphism of the huia (*Heteralocha acutirostris*): sexual selection or intersexual competition.. Notornis.

[13] Rice WR (1989). Analyzing tables of statistical tests.. Evolution.

[14] Paetkau D (2003). An empirical exploration of data quality in DNA-based population inventories.. Molecular Ecology.

[15] Creel S, Spong G, Sands JL, Rotella J, Zeigle J (2003). Population size estimation in Yellowstone wolves with error-prone noninvasive microsatellite genotypes.. Molecular Ecology.

[16] Manel S, Gaggiotti OE, Waples RS (2005). Assignment methods: matching biological questions with appropriate techniques.. Trends in Ecology and Evolution.

[17] Broquet T, Petit E (2004). Quantifying genotyping errors in noninvasive population genetics.. Molecular Ecology.

[18] Hung C-M, Li S-H, Lee LL (2004). Faecal DNA typing to determine the abundance and spatial organisation of otters (*Lutra lutra*) along two stream systems in Kinmen.. Animal Conservation.

[19] Double M, Murphy S (2000). Genetic variation within and among populations of North Island kokako. Science and Research Internal Report 176..

[20] Armstrong DP, Davidson RS, Perrott JK, Roygard J, Buchanan L (2005). Density-dependent population growth in a reintroduced population of North Island saddlebacks.. Journal of Animal Ecology.

[21] Millar CD, Dodd A, Anderson J, Gibb GC, Ritchie PA (2008). Mutation and evolutionary rates in Adélie penguins from the Antarctic.. Public Library of Science Genetics.

[22] Frankham R (1995). Effective population size/adult population size ratios in wildlife: a review.. Genetical Research.

[23] Wenink PW, Baker AJ, Tilanus MG (1994). Mitochondrial control-region sequences in two shorebird species, the turnstone and the dunlin, and their utility in population genetic studies.. Molecular Biology and Evolution.

[24] Lambert DM, King T, Shepherd LD, Livingston A, Anderson S (2005). Serial population bottlenecks and genetic variation: translocated populations of the New Zealand saddleback (*Philesturnus carunculatus rufusater*).. Conservation Genetics.

[25] Ellegren H, Primmer CR, Sheldon BC (1995). Microsatellite ‘evolution’: directionality or bias.. Nature Genetics.

[26] Grosler AG, Blondel J, Gosler A, Lebreton JD, McCleery R (1990). The variable niche hypothesis revisited: an analysis of intra- and inter-specific differences in bill variation in *Parus*.. Population Biology of Passerine Birds: an Integrated Approach.

[27] Grosler AG, Carruthers TD (1994). Bill size and niche breadth in the Irish coal tit *Parus ater hibernicus*.. Journal of Avian Biology.

[28] Hudson QJ, Wilkins RJ, Waas JR, Hogg ID (2000). Low genetic variability in small populations of New Zealand kokako *Callaeas cinerea* wilsoni.. Biological Conservation.

[29] Innes J, Flux I (1999). North Island recovery plan 1999-2009. Threatened Species Recovery Plan 30..

[30] Pritchard JK, Stephens M, Donnelly P (2000). Inferences of population structure using multilocus genotype data.. Genetics.

[31] Dawson KJ, Belkhir K (2001). A Bayesian approach to the identification of panmictic populations and the assignment of individuals.. Genetical Research.

[32] Griffiths R, Double MC, Orr K, Dawson JG (1998). A DNA test to sex most birds.. Molecular Ecology.

[33] Hudson QJ (1999). Genetic variation within and among populations of kokako (*Callaeas cinerea*)..

[34] Navidi W, Arnheim N, Waterman MS (1992). A multiple-tube approach for accurate genotyping of very small DNA samples by using PCR: statistical considerations.. American Journal of Human Genetics.

[35] Taberlet P, Griffin S, Goossens B, Questiau S, Manceau V (1996). Reliable genotyping of samples with very low DNA quantities using PCR.. Nucleic Acids Research.

[36] Raymond M, Rousset F (1995). GENEPOP (version 1.2): population genetics software for exact tests and ecumenicism.. Journal of Heredity.

[37] Gagneux P, Boesch C, Woodruff DS (1997). Microsatellite scoring errors associated with noninvasive genotyping based on nuclear DNA amplified from shed hair.. Molecular Ecology.

[38] Falush D, Stephens M, Pritchard JK (2003). Inference of population structure using multilocus genotype data: linked loci and correlated allele frequencies.. Genetics.

[39] Pearse DE, Crandall KA (2004). Beyond Fst: analysis of population genetic data for conservation.. Conservation Genetics.

[40] Kuhner MK (2006). LAMARC (version 2.0): maximum likelihood and Bayesian estimation of population parameters.. Bioinformatics.

